# Why *in vivo* models of disease remain indispensable

**DOI:** 10.1242/dmm.052997

**Published:** 2026-05-05

**Authors:** Debora Bogani, Kirsty Hooper, Owen Sansom

**Affiliations:** ^1^ Mary Lyon Centre at MRC Harwell, Harwell Campus, Didcot OX11 0RD, UK; ^2^ The Company of Biologists, Bidder Building, Station Road, Histon, Cambridge CB24 9LF, UK; ^3^ CRUK Scotland Institute, Glasgow G61 1BD, UK; ^4^ School of Cancer Sciences, University of Glasgow, Glasgow G61 1QH, UK

## Abstract

**Summary:** The call to arms for alternatives to model organisms of disease will fail without ongoing integration with – and validation against – complex animal models and support from the animal modelling community.

## Editorial preface

Biomedical research is entering a period of accelerated methodological and regulatory change. In the UK, the government has published a cross-sector strategy to support the development and uptake of non-animal approaches in life sciences research. In parallel, the U.S. Food and Drug Administration (FDA) has announced policy changes aimed at expanding regulatory acceptance of non-animal methods for specific preclinical applications. Together, these announcements signal a growing international consensus that innovation in research methods must keep pace with scientific capability, ethical expectations and patient need.

These policy developments have been widely welcomed, reflecting the fact that researchers in the UK already adhere to established animal research principles such as the 3Rs (the ‘Replacement, Reduction and Refinement’ of animals used in research). Although these new policies have attracted headlines that suggest an accelerated move away from *in vivo* models, closer examination of the data behind the policy documents highlights that these alternative approaches remain far from recapitulating the complexity of the diseases that are likely to pose the greatest challenges to human health over the next 50 years. The UK governmental policy paper acknowledges that many complex disease processes cannot yet be modelled adequately in alternative systems, and that *in vivo* models will continue to be required both to understand these diseases and to develop new therapies (baskets 2 and 3 in the documents). It is, therefore, important that, at a time of limited funding, efforts to increase investment in alternative approaches do not lead to disinvestment in complex *in vivo* models of disease. Moreover, the *in vivo* disease-modelling community is at the forefront of adopting emerging technologies, including gene and base editing, environmental perturbation, spatial biology, and advances in data science and artificial intelligence (AI), all of which will be essential in assessing the predictive value of new disease models.

These new policies, therefore, raise important questions for the research community and its stakeholders. How should emerging alternative approaches be integrated responsibly into discovery and development pipelines? What evidence is required to determine when approaches can replace animal studies, and when *in vivo* models remain essential, particularly for safety assessment ahead of clinical trials? How can policy frameworks avoid creating false dichotomies that risk undermining scientific robustness or translational confidence?

**Fig. 1.**
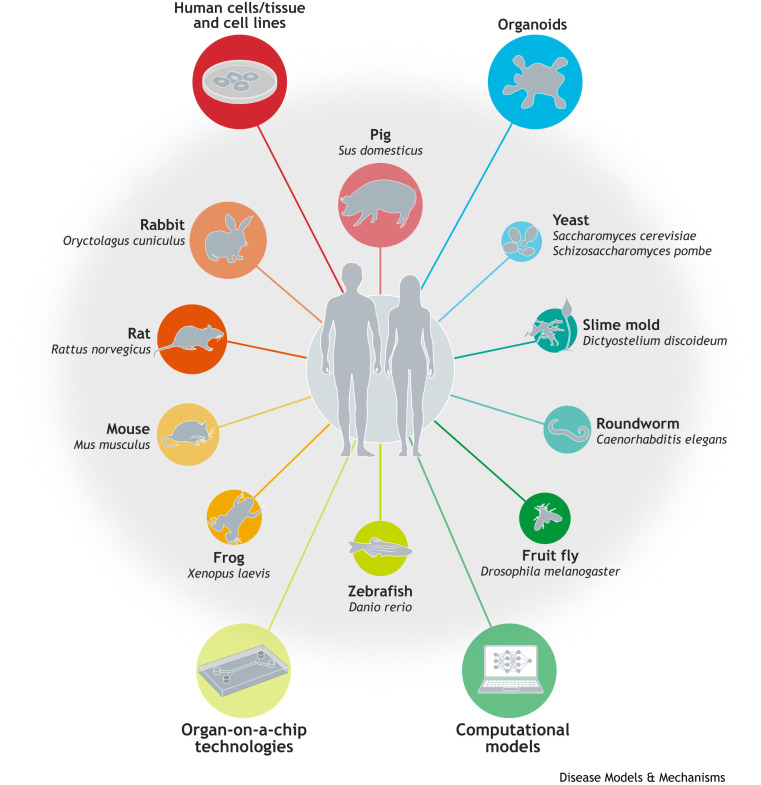
A spectrum of model systems used to study human disease.

This Editorial brings together perspectives from communities across UK bioscience and beyond, through the MRC National Mouse Genetics Network (NMGN) and the Editors of Disease Models & Mechanisms (DMM; Box 1), to argue for a balanced, evidence-led approach. It highlights not only the continued importance of *in vivo* models – particularly genetically refined mouse models – but also the crucial and often underappreciated role that the animal research community has played in developing, validating and funding the very alternative models now shaping policy discussions. Rather than framing the future as a choice between animal models and alternatives, we argue that progress will depend on their strategic integration within a cohesive research ecosystem (Fig. 1).

Box 1. Views from cross-disciplinary DMM Editors At DMM, we asked Editors for their views on current model systems and their applicability to their respective research areas. A clear consensus emerged: all are using, developing or supportive of novel alternative models, but key critical aspects of disease biology are still missing. A selection of their comments is presented below.

**Sally Dunwoodie (Victor Chang Cardiac Research Institute, Darlinghurst, Australia)**

My research focuses on identifying genetic and/or environmental causes of congenital malformation in humans. We use mice to study human development as it is a robust model due to genetic relatedness, conservation of developmental processes and of embryo–fetal form and function, and comparable physiological processes. Human induced pluripotent stem cell (iPSC) and various differentiation protocols are being developed as an approximation of early human embryo development. These can model some aspects of early embryogenesis, or the development of a particular structure or organ, and some are providing important molecular and cellular insights into human development. Given the complexity of embryogenesis, *in vivo* models are required to recapitulate its multi-dimensional interdependent processes and influences, including the maternal–fetal environment. This is not the case for all areas of research relevant to human. Therefore, rather than criticising all animal experiments with a view to banning them, we should identify those diseases and research areas that need to be modelled *in vivo*.

Australia's regulatory landscape for *in vivo* research is tightening, driven by ethical considerations and global alignment. The Australian code for the care and use of animals for scientific purposes is being reviewed. Generally, the Therapeutic Goods Administration adopts international scientific guidelines, following consultation.

**Vivian Li (The Francis Crick Institute, London, UK)**

The central focus of our laboratory is to uncover the mechanisms governing Wnt regulation in homeostasis, regeneration and colorectal cancer. We integrate genetically engineered mouse models, patient-derived organoids and advanced genomic tools to investigate how Wnt signalling and cell-state plasticity contribute to homeostasis and disease, particularly therapy resistance in colorectal cancer. We have also pioneered organoid-based regenerative strategies that combine patient-derived organoids with biomaterial scaffolds to engineer human intestinal grafts. Since the establishment of organoid culture systems in 2009, this technology has transformed research in stem cell and cancer research. The rapid and self-sustaining growth capacity of organoids makes them an ideal cell source for regenerative medicine. Their ability to expand indefinitely while maintaining tissue identity enables the generation of clinically relevant cell numbers, supporting applications such as tissue repair, transplantation and disease modelling in a way previously unattainable with primary cells. Patient-derived organoids now serve as powerful preclinical platforms that faithfully recapitulate tumour heterogeneity and treatment responses, enabling personalised drug testing, mechanistic discovery and the development of more predictive therapeutic strategies.

Although organoids represent powerful *in vitro* platforms for disease modelling, they do not fully capture the complexity of the tumour microenvironment present *in vivo*. Co-culturing systems incorporating stromal or immune components can partially reconstitute specific cellular interactions but cannot recapitulate the integrated, dynamic and systemic responses of a living organism. Tissue explants preserve aspects of native architecture and microenvironmental cues, yet are constrained by limited size, viability and culture duration. Therefore, *in vivo* tumour models remain indispensable for investigating tumour evolution and therapeutic responses within an intact immune and vascular system. Together, advanced organoid platforms and *in vivo* models constitute a complementary and translational pipeline, essential for bridging fundamental biological discoveries to effective clinical therapies.

**Karen Liu (King's College London, London, UK)**

We primarily do comparative analysis using human stem cells/organoids and genetically modified mouse models, with some minor use of frog models. We use this approach because, for developmental anomalies, it is impossible to assess true function without *in vivo* models. For our research, the best rationale for using an alternative model is when one has access to patient tissues/cells. However, patient cells are inherently variable, and often there are no suitable controls. Therefore, one is balancing patient specificity/relevance versus experimental rigour. In my opinion, these must go hand in hand: using human cell models to point us in the right direction, while using animal models for the best, most rigorous, reproducible experiments. More recently, computational models have been used to generate predictions. This is a great move as we need to deal with big data; however, this still needs grounding in real-life biology and needs robust experimentation to test the predictions. Furthermore, future use of machine learning will only be as good as the baseline ‘real’ data. Finally, we often don't explain that it would take 100s/1000s of ‘alternative’ experiments to replicate one single mouse model.

**Sumana Sanyal (University of Oxford, Oxford, UK)**

My laboratory investigates how dengue and zika viruses build infectious particles within host cells and dysregulate immune responses. We use human cell lines and iPSC-derived models that mimic hepatocytes, monocytes and neural cells. iPSC-derived models and organoids have been transformative. They enable us to study viral infection in physiologically relevant human cell types without requiring tissue from patients. As our research focuses on how viruses reorganise subcellular structures within cells, or target immune signalling pathways, these experimental models work exceptionally well for answering our questions at a molecular level. For dengue and zika virus research, where different tissues respond differently to infection, having consistent supplies of liver, immune and neural cells has enabled us to discover virus-induced compartments and immune alterations that drive tissue-specific pathology. When we identify genes essential for viral particle production in cells, mouse studies confirm whether these pathways matter during infection at a whole-organism level. Animal models remain essential for validating therapeutic targets, understanding viral evolution within hosts and detecting toxicity invisible in cell culture. While the regulatory framework has improved research standards, continued discussion about proportionality would be valuable, particularly for neglected tropical diseases, for which immunopathology is central to disease and alternatives remain limited.

**Ian Smyth (Monash University, Melbourne, Australia)**

We principally use genetically modified mouse models to study kidney disease and for efficacy testing for newly developed potential therapies. While kidney organoids have developed significantly in the last few years, they remain poor models of the organ *in vivo*. They lack a circulatory system and immune cells (which are often implicated in disease processes). The kidney has upwards of 40 renal-specific cell types, with another 30 or so resident in the organ but from other places (immune cells, etc.). That type of cellular diversity does not exist in organoids, which themselves are very much fetal in nature (first trimester or so). *In vivo* models are best suited in terms of matching the complex and dynamic physiology associated with the kidney, which acts as an interface with the cardiovascular system. Although mice are clearly not humans, and they differ in some regards, there is a good correlation between equivalent genetic variants in mouse and human. There certainly exists scope for cellular systems to help refine animal use in this context, but whether this means fewer animals are used or that they are just better used, remains an open question.

**David Tobin (Duke University School of Medicine, Durham, NC, USA)**

We rely heavily on animal models to understand the genetics of *Mycobacterium tuberculosis* and human susceptibility to tuberculosis (TB). *In vitro* and *ex vivo* profiling can give us important insights, but TB involves so many heterogeneous local, systemic, and spatially organised immune responses and bacterial–host and multicellular interactions that causality of any bacterial or host variant becomes very hard to establish rigorously without being able to access whole animals. The field has used explant models from infected animals or *in vitro* models that assemble central immune structures called granulomas from human immune cells, and these have been useful tools for examining specific aspects of infection, but these alternatives don't recreate the entirety of the host immune response or even the diverse niches in which we know the bacteria reside in humans and in animal models. Deprioritising *in vivo* models and model organisms – which have provided foundational insights into both disease and fundamental concepts in biology – limits or eliminates many of our most useful approaches for rigorously assessing causality, both genetic and therapeutic.

## Integrating animal models and alternatives: shaping the future of preclinical research

Debates about animal research and alternatives are often framed as a contest between old and new approaches. This framing is both misleading and counterproductive. In practice, modern biomedical research is increasingly characterised by methodological pluralism, with animal models, human cell-based systems, organoids, organ-on-a-chip technologies and computational approaches used side by side to address different biological questions.

Importantly, many of the most celebrated alternative models have emerged not in opposition to animal research but from within it. Breakthroughs in stem cell biology, organoid culture and computational modelling have been driven largely by researchers trained in *in vivo* biology and informed by decades of animal research. Several therapeutic concepts now being pursued in human-based systems were first validated in animal models, including interventions targeting ageing (Baker et al., 2011; Conboy et al., 2005), senescence (Baker et al., 2011) and genetic compensation mechanisms (Baker et al., 2011; Rossi et al., 2015). Mouse models, in particular, have revealed mechanisms underlying ageing and systemic decline – such as the accumulation of senescent cells and the role of circulating factors – that later became targets for therapeutic intervention (Baker et al., 2011; Conboy et al., 2005).

In a recent liver cancer study, researchers developed a suite of organoid models and complex mouse models and compared them with human liver cancer samples to identify new tumour subtypes and potential therapeutic strategies (Müller et al., 2025). Importantly, mouse and human tumours of the same subtype were more closely related than human tumours of different subtypes, and the mouse subtype tumours recapitulated patient responses to standard-of-care (SOC) treatments. Organoid screens were valuable for identifying new therapeutic combinations targeting the tumour epithelium, but they were unable to reproduce SOC responses. Ultimately, the clinical trial that will follow on from this work will, therefore, require the integration of *in vivo* models, *in vitro* organoids and human samples.

## Alternative models that do well and their persistent scientific limitations

Non-animal approaches have already delivered major benefits. Human cell cultures and induced pluripotent stem cell (iPSC)-derived systems allow the direct interrogation of disease-associated variants in a human genetic context. Organoids capture aspects of three-dimensional tissue organisation that are inaccessible in traditional monolayer cultures. Organ-on-a-chip technologies introduce controlled mechanical forces and microfluidics, while computational and AI-based approaches integrate large datasets and generate new hypotheses (Deng et al., 2023; Obeid et al., 2024).

Critically, the design and interpretation of these systems rely heavily on biological knowledge derived from animal research, including principles of development, tissue architecture, immune signalling and systemic physiology. Much of this foundational understanding comes from decades of work in genetically tractable animal models. Without this foundation, alternatives risk becoming technically sophisticated but biologically incomplete.

Despite rapid progress in this area, significant scientific limitations remain that prevent the replacement of animal models. Most alternative systems struggle to reproduce systemic context: interactions between organs, endocrine signalling, immune surveillance, vascular perfusion, metabolism, cachexia, ageing and long-term adaptation (Ferrer et al., 2023). These features are central to many diseases and to testing the safety and efficacy of therapies.

For example, in cancer research, organoids and *ex vivo* cultures have proven powerful for identifying oncogenic pathways and tumour cell states (Qin et al., 2023). However, immune surveillance and immune evasion, both key determinants of tumour initiation and progression, often still require *in vivo* models. Indeed, the mechanisms underlying the loss of γδ T cell-mediated tumour surveillance in colorectal cancer could only be resolved through integrated studies combining mouse models, human tumour tissue and complementary *in vitro* systems (Suzuki et al., 2023). Another important limitation of these *in vitro* systems is their inability to replicate a representative tumour microenvironment.

In Down syndrome-associated Alzheimer's disease, human cellular models and iPSC-derived systems have provided important insights into cell-type-specific pathology (Wu et al., 2023, 2022). However, they cannot capture whole-organ and systemic risks, such as treatment-associated cerebral bleeding, which currently limit the use of emerging Alzheimer's disease therapies in people with Down syndrome (Farrell et al., 2025; Rafii et al., 2025).

Reproducibility and biological stability can also present important challenges. Organoid systems can vary widely between laboratories, and donor-to-donor variability remains high, although aspects of such variability can be harnessed to better represent population and patient heterogeneity. Human pluripotent stem cell models can acquire genetic and epigenetic abnormalities over time, complicating interpretation and comparability, particularly in studies of sex differences and long-term disease processes (Merkle et al., 2017). Reproducibility is also a known issue affecting experimental results obtained using animal models, which is why a combinatorial approach is the most effective way to offset the limitations of each individual model system.

## Why *in vivo* models remain indispensable

Against this background, *in vivo* animal models remain essential in many areas of biomedical research. Their value lies not in perfectly replicating human disease, but in enabling experiments at the level of the intact organism. Immune responses, developmental processes, sex differences, ageing, pharmacokinetics and off-target toxicity all depend on interactions that cannot yet be reproduced outside a living system.

For instance, *in vivo* functional genomics studies have shown that perturbations producing clear effects *in vitro* can behave very differently within the physiological context of an organism, particularly in metabolic and differentiation pathways (Lara-Astiaso et al., 2023). Furthermore, *in vivo* CRISPR screens have highlighted the importance of the intact immune system in shaping tumour evolution and therapeutic response, interactions that are difficult to recapitulate *in vitro* (Martin et al., 2021).

It is also important to note that many diseases co-occur, and the systemic impacts of disease are expansive. Complex factors such as the impact of obesity and metabolic disease on offspring (Godfrey et al., 2017) are extremely difficult to model *ex vivo*. Furthermore, it is now easier than ever to modify environmental factors or examine co-morbidity in *in vivo* models and to compare the results with human disease tissues and cohort studies (Swanton et al., 2024). This approach generates rich datasets for AI and machine-learning tools, including those used in digital twin studies.

These considerations are increasingly important in the era of AI. Although AI and machine learning offer powerful tools for hypothesis generation and data integration, their predictive accuracy relies on the availability of large, high-quality biological datasets. Studies evaluating *in silico* predictions of pathogenicity demonstrate that computational approaches alone remain insufficiently reliable for clinical decision making without biological validation (Thusberg et al., 2011; Amendola et al., 2015). At present, such validation depends heavily on *in vivo* data.

## Drug discovery and development still require integrated approaches

The broader pharmaceutical industry has invested significantly in 3Rs initiatives, leading to a substantial shift in how animal models are used in drug discovery. Alongside the 3Rs, the need to reduce the time and cost of drug development has created strong incentives to improve competitiveness and achieve more with fewer resources. Early replacement of animals with *in vitro* and even *in silico* predictive systems has been adopted in the drug metabolism and pharmacokinetics (DMPK) discipline. Where compounds were previously screened *in vivo* using rapid pharmacokinetic assays, *in vitro* assays are now increasingly used to filter candidates before they progress to *in vivo* assessment; in cases in which the drug template information is well established, predictive modelling can also guide the drug discovery process (Mak et al., 2022; Williamson et al., 2020). Nevertheless, *in vitro* systems do not fully recapitulate systemic drug perfusion, absorption, tissue distribution and clearance, all of which are essential considerations in the development of new drug classes. Accurate DMPK assessment and dose prediction are essential for safety and success in early clinical trials, and animal models, alongside alternative systems, are still required to achieve this.

Cancer treatments, among others, are increasingly combinatorial. Previously, combination strategies could often be developed empirically in the clinic, with testing of chemotherapy cocktails driving changes in SOC therapy. However, the questions now being asked and the therapies now being developed are far more complex. With improvements in SOC therapy and innovation in treatment modalities, drug development has moved beyond monotherapies based on small molecules, antibodies or chemotherapy towards four- or five-drug cocktails that often include novel platforms, such as CAR-T therapies for solid tumours, tumour-targeted immune engagers, antibody–drug conjugates, protein degraders, molecular glues and RNA-based drugs. Cell-panel testing revolutionised our ability to assess monotherapy (Corsello et al., 2017; Rauner et al., 2025) and, indeed, combination treatments across different tumour types (Bashi et al., 2024). More recently, tumour organoids and even tissue slices (Kenerson et al., 2021; Trivedi et al., 2024) have provided assay systems that recapitulate some aspects of human disease and have enhanced our ability to evaluate drug combinations. Reductionist approaches have resulted in *ex vivo* assays that incorporate elements of the immune system to help answer complex questions and more adequately assess immune-based cancer therapies (Wang et al., 2025a,b). Combination therapies, especially those involving complex novel treatments, require precise control of scheduling, dosing and sequence of administration. Although *in vitro* systems have been valuable for early triaging, *in vivo* systems remain necessary to establish these parameters, which are underpinned by important factors – such as absorption, pharmacokinetic profile and tissue distribution – before therapies can progress to human clinical trials.

## The MRC NMGN as an integrative platform

Within this evolving ecosystem, the NMGN exemplifies how animal research can align with modern expectations of rigour, efficiency and integration. Funded by UKRI–MRC, the NMGN brings together broad expertise across multiple model systems, focusing on key health and technological challenges, and applying the most appropriate approach in each case. By focusing on precision genetic models, shared infrastructure and open data, the Network seeks to maximise the translational relevance of *in vivo* studies while minimising unnecessary duplication.

Crucially, the outputs of networks such as the NMGN, including deep phenotyping studies, underpin the development and validation of alternative approaches. Given that *in vitro* and computational systems frequently depend on well-characterised mouse data to establish biological relevance, define limitations and guide interpretation, excellence in animal genetics is not in tension with the development of alternatives but foundational to their success.

## Who funds and drives alternatives?

An often overlooked feature of the current landscape is that the largest funders and adopters of non-animal methods are institutions that also fund or conduct animal research. Public agencies, pharmaceutical companies, contract research organisations and national 3Rs centres simultaneously invest in alternatives while maintaining *in vivo* capability.

In the UK, public funders support non-animal technologies through dedicated programmes while continuing to invest in animal-based discovery science. Internationally, large-scale initiatives to develop organ-on-a-chip and microphysiological systems are embedded within research ecosystems that continue to rely on *in vivo* data for validation and regulatory confidence. Pharmaceutical companies and contract research organisations – among the biggest investors in organoids, organ-on-a-chip systems and computational tools – deploy these approaches primarily to refine hypotheses, reduce attrition and improve the design of *in vivo* and clinical studies. Taken together, these demonstrate that our varied research ecosystems continue to depend on a combinatorial approach to modelling disease.

## Policy implications and the path forward

Recent UK and FDA policy signals represent an opportunity to modernise preclinical research. To realise this opportunity, policy frameworks must avoid presenting alternatives as wholesale substitutes for animal models across all contexts. Instead, they should prioritise strategic integration, rigorous benchmarking against validated *in vivo* and clinical data, sustained investment in shared infrastructure and regulatory clarity on where alternatives can replace, reduce or refine animal studies.

The future of preclinical research will not be defined by a simple transition from animal models to alternatives. It will instead be shaped by how effectively the scientific community integrates diverse approaches to address the complexity of human disease. Journals also have a critical role to play: balanced reporting of both the strengths and limitations of all model systems – animal and non-animal alike – will be essential to prevent hype from outpacing evidence.

By recognising this interdependence, and by supporting initiatives such as the MRC NMGN alongside sustained investment in non-animal technologies, it is possible to deliver research that is more predictive, more ethical and more impactful. In this integrated future, animal models are not an obstacle to progress but a foundation upon which better alternatives, better data and better translation can be built.
